# Comparative Analysis of the Growth, Physiological Responses, and Gene Expression of Chinese Soft-Shelled Turtles Cultured in Different Modes

**DOI:** 10.3390/ani14060962

**Published:** 2024-03-20

**Authors:** Benli Wu, Long Huang, Cangcang Wu, Jing Chen, Xiajun Chen, Jixiang He

**Affiliations:** Anhui Province Key Laboratory of Aquaculture and Stock Enhancement, Fisheries Research Institute, Anhui Academy of Agricultural Sciences, Hefei 230031, China; wubenli5555@163.com (B.W.);

**Keywords:** rice–turtle co-culture, pond culture, growth performance, physiological response, gene expression

## Abstract

**Simple Summary:**

Different culturing modes have adopted different living spaces, foods, shelter, substrates, and surroundings for and human actions regarding animals. The Chinese soft-shelled turtle (*Pelodiscus sinensis*) is one of most economically important turtles, and healthier culturing modes should be applied for high-quality aquatic products to remit serious environmental and health problems. The differences in growth performance, physiological indices, and related gene expression were compared between two culturing modes in the present study. The results indicated that rice–turtle-co-cultured turtles with limited natural foods grew slower than pond-cultured turtles fed with commercial feeds. The creatinine, uric acid, blood urea nitrogen, alkaline phosphatase, acid phosphatase, glutamic oxaloacetic transaminase, and catalase were higher in co-cultured turtles than pond-cultured turtles. More differentially expressed genes were related to environmental information processing, metabolism, and human diseases when comparing pond-cultured turtles and co-cultured turtles. *P. sinensis* can adapt to different culture modes by adjusting their growth and physiology over a short culture period, and rice–turtle co-culturing proves to be a healthier culturing mode for turtles within the proper culturing strategies.

**Abstract:**

The Chinese soft-shelled turtle (*Pelodiscus sinensis*) is an important freshwater aquaculture turtle due to its taste and nutritional and medicinal value. More ecological culturing modes, such as rice–turtle co-culture, should be developed to meet the ecological benefit demand. We compared growth, physiological parameters, and transcriptome data to detect the physiological responses and regulatory mechanisms of pond-cultured turtles as compared to co-cultured turtles. The co-cultured turtles grew slower than pond-cultured turtles. The gonadosomatic index of co-cultured male turtles was lower than that of pond-cultured male turtles, and both the mesenteric fat index and limb fat index were lower in co-cultured turtles than in pond-cultured turtles (*p* < 0.05). The blood GLU of the co-cultured turtles was significantly lower than the GLU of the pond-cultured turtles (*p* < 0.05), while the values of CRE, UA, BUN, AKP, ACP, GOT, and CAT were higher in the co-cultured turtles than in the pond-cultured turtles (*p* < 0.05). In total, 246 and 598 differentially expressed genes (DEGs) were identified in the brain and gut from turtles cultured in the two different modes, respectively. More DEGs were related to environmental information processing, metabolism, and human diseases. In the brain, the top enriched pathways of DEGs included the longevity regulating pathway, glycerolipid metabolism, cytokine–cytokine receptor interaction, Toll-like receptor signaling pathway, and PI3K-Akt signaling pathway, while in the gut, the top enriched pathways of DEGs included the cell cycle, DNA replication, cellular senescence, and p53 signaling pathway. The turtles acclimated to the different culturing conditions by adjusting their growth, physiological, and biochemical characteristics and related gene expression during a short culture period.

## 1. Introduction

The wild resources of turtles face increasing ecological threats, such as habitat destruction and environmental pollution [1,2]. The Chinese soft-shelled turtle (*Pelodiscus sinensis*) has been an important freshwater aquaculture reptile for a long time in East Asia due to its desirable taste, nutritional and medicinal value, and the scarcity of its wild resources. It is the one of most economically important turtles, with millions of them being farmed in China every year [3]. Commonly, *P. sinensis* are intensively cultivated in greenhouses or artificial ponds with high stocking density and are provided excessively artificial diets to achieve fast growth and high production [4,5]. Captive culture has significantly increased the production of commercial turtles, but it is also accompanied by problems such as a high risk of disease, defective appearance, and low quality, which cannot meet the customers’ increasing pursuit of food quality [6,7]. Furthermore, high-energy consumption rearing patterns are not conducive to sustainable agricultural development and have aroused increasing public concern. Healthier culturing modes should be applied for high-quality aquatic products to remit serious environmental and health problems that affect both the quality of the product and animal welfare [8,9].

Different habitats provide different living spaces, foods, shelter, substrates, and surroundings for as well as human actions regarding animals [10,11]. Turtles have been found to experience less stress in and showed a distinct preference for an enriched environment [12,13] and may have different growth rates or metabolic rates depending on the availability of resources or the presence of stressors [14]. *P. sinensis* is an opportunistic predator; in different habitats, it will make adaptive responses in behavior, growth, physiology, and metabolism [15,16]. Growth is a highly complex process that involves the regulation of appetite, muscle growth, and weight gain [17], which is affected by genetic factors, initial body size, age, sex, and culture conditions [18,19].

Integrated agri–aquaculture systems such as rice–crayfish, rice–fish, and rice–turtle have expanded rapidly due to their ecological efficiency and contributions to food productivity and security in recent years [20,21,22]. Rice–turtle co-culturing is a stable and ecological co-culturing mode that can effectively alleviate the quality and environmental problems caused by wild resource attenuation and intensive breeding [23,24]. *P. sinensis* is amphibious, and wild turtles are aggressive and mainly carnivorous. Turtles can adapt to different habitats, and divergence in growth, appearance, and gut microbes can be observed within a relatively short culture duration [25].

The goal of rice–turtle co-culturing is to reduce costs, increase efficiency, protect the environment, preserve the wild habits of turtles, and improve the quality of cultured turtles as much as possible [24]. The feeding of natural bait, such as small fish and shrimp, benthic animals, and aquatic insects, in paddy fields can reduce nutrient consumption and control the occurrence of diseases and insect pests [26]. Moreover, commercial feeds should be limited to those provided for turtles cultured in paddies, which could minimize nutrient outputs and decrease the environmental impacts of intensive culture [27]. Furthermore, co-cultured turtles have proven to yield better flavor, and their appearance is more accepted by consumers [3].

However, natural food is also limited in paddy fields for the fast growth of turtles, and culture strategies involving stocking density, feeding regime, disease control, and environmental conservation should be further optimized for economic and ecological benefits. Physiological and biochemical indices can reflect the health status of turtles, and transcriptome analysis of brain and gut tissues would also help to further reveal key genes and pathways related to the growth and physiological adaptation of turtles cultured in different modes. Systematic studies on the adaptive process of turtles in different culture habitats would provide a basis for optimizing ecological farming strategies and promoting animal welfare.

## 2. Materials and Methods

### 2.1. Rice Planting and Turtle Rearing

The experimental paddy fields were located in Taihu County, Anhui Province, China (E 116°21′, N 30°18′). Chinese soft-shelled turtles (Japanese strain) were held in cement tanks in a greenhouse before being allocated to pond and paddy field plots. The initial body weight was 756.3 ± 16.7 g. The experimental ponds were approximately 2000 m^2^ and 1.5 m deep (group marked as PT). The experimental paddy fields were also approximately 2000 m^2^, and the area was modified for turtle cultivation with a 200 m^2^ pond (1.5 m deep) on the side (group marked as RT). The depth of water in the rice field was 0–20 cm during the experiment, which meet the needs of rice growth. The stocking densities were 1000 turtles/pond and 100 turtles/paddy, which have been commonly adopted in turtle culture in previous studies. Triplicate ponds and paddies were monitored; in total, 3300 turtles were cultured in the experiment (♀:♂ = 1:1).The pond-cultured turtles were fed a commercial feed that contained 43% crude protein (Jinjia, Hangzhou, China) twice daily at 09:00 a.m. and 16:00 p.m., and the daily feeding level was 4% during the experiment. The co-cultured turtles were not fed commercial diets; instead, some small live fish and winkles from the surrounding water were supplemented during the trial. The mode was considered more ecological and wilder than actual farming. The growing period of rice was from 15 June to 16 October, and the turtle-culturing experiment lasted from 1 July to 30 September in 2022.

### 2.2. Measurement and Sampling

The trial lasted 90 days, and as many turtles as possible were collected for measurement. The sex was identified and measured separately based on appearance differences. Every third turtle with no visible trauma from the pond and paddies was anesthetized and euthanized by intramuscular injection (tiletamine:zolazepam = 1:1, 30 mg/kg, Zoletil^®^50, Virbac, Carros, France). The turtles were quickly decapitated in an unconscious state, blood was collected in tubes with heparin sodium and centrifuged (4000 r, 10 min, Eppendorf, Hamburg, Germany), and the supernatant plasma was stored at −80 °C before biochemical analysis. The liver, gut, limb fat, and mesenteric fat were carefully removed, placed on ice, and weighed. The liver, intestine (gut), and brain tissues were flash-frozen in liquid nitrogen for physiological, biochemical, and transcriptome sequencing analysis. All operations on turtles were conducted in accordance with the institutional animal care guidelines and the supervision of Anhui Academy of Agricultural Sciences committees (NO.AAAS 2021-12).

The parameters measured and the corresponding calculations were as follows:

- Specific growth rate (SGR, %/d) = 100 × (lnFBW − lnIBW)/days;
- Hepatosomatic index (HSI, %) = 100 × liver weight/body weight;
- Gonadosomatic index (GSI, %) = 100 × gonad weight/body weight;
- Intestinal somatic index (ISI, %) =100 × intestinal weight/body weight;
- Mesenteric fat index (MFI, %) = 100 × mesenteric fat weight/body weight;
- Limb fat index (LFI, %) = 100 × limb fat weight/body weight.

IBW and FBW indicate initial body weight and final body weight, respectively.

### 2.3. Physiological and Biochemical Parameter Analysis

The biochemical parameters of plasma, including protein (TP), glucose (GLU), triglyceride (TG), total cholesterol (TCHO), blood urea nitrogen (BUN), uric acid (UA), creatinine (CRE), superoxide dismutase (SOD), catalase (CAT), glutathione peroxidase (GSH-PX), alkaline phosphatase (AKP), acid phosphatase (ACP), glutamic-pyruvic transaminase (GPT), glutamic oxaloacetic transaminase (GOT), lysozyme (LZM), and the activities of protease, lipase, and amylase were determined using commercial kits (Jiancheng Biotech. Co., Nanjing, China). A fish complement 3 (C3) ELISA kit was used for C3 determination (Baolai Biotech. Co., Yancheng, China).

### 2.4. RNA Extraction and Transcriptome Sequencing Analysis

Total RNA of brains and guts (three replicate samples for each tissue) from male turtles cultured in ponds and paddy fields was extracted using a TRIzol reagent kit (Invitrogen, Carlsbad, CA, USA) according to the manufacturer’s protocol. The samples of pond-cultured turtle brain and gut were marked as PTB and PTG, and samples of co-cultured turtle brain and gut were marked as RTB and RTG, respectively. RNA quality was assessed on an Agilent 2100 Bioanalyzer (Agilent Technologies, Palo Alto, CA, USA) and checked using RNase-free agarose gel electrophoresis. After total RNA was extracted, eukaryotic mRNA was enriched by Oligo(dT) beads (Invitrogen, Shanghai, China). Then, the enriched mRNA was fragmented into short fragments using fragmentation buffer and reverse-transcribed into cDNA by using the NEBNext Ultra RNA Library Prep Kit for Illumina (NEB #7530, New England Biolabs, Ipswich, MA, USA). The purified double-stranded cDNA fragments were end-repaired, a base was added, and the fragments were ligated to Illumina sequencing adapters. The ligation reaction was purified with AMPure XP Beads (1.0×) (Beckman Coulter, Brea, CA, USA). Ligated fragments were subjected to size selection by agarose gel electrophoresis and PCR amplification. The resulting cDNA library was sequenced using Illumina NovaSeq 6000 by Gene Denovo Biotechnology Co. (Guangzhou, China).

The bioinformatics analysis included filtering clean reads, alignment with rRNA, alignment with the reference genome of Chinese soft-shelled turtles (PelSin_1.0, http://ftp.ensembl.org/pub/release-78/fasta/pelodiscus_sinensis/dna/, URL accessed on 17 November 2014), and quantification of gene abundance. The differential gene analysis was conducted by using DESeq2, FDR value ≤ 0.05. Then, the differentially expressed genes (DEGs) were subjected to KEGG pathway enrichment analysis. The raw sequence data were submitted to the National Center for Biotechnology Information (NCBI) Sequence Read Archive (SRA) (accession number: PRJNA971802).

### 2.5. Gene Expression Validation by qPCR

To validate the RNA-seq data and gene expression profiles, every 10 DEGs were selected to perform qPCR for the brain and gut. Specific primers were designed using Primer 5 based on the coding sequences of identified genes from the turtle genome, and the sequences of primers are listed in Supplementary Table S1. qPCR was performed using the LightCycler^®^480 Real-Time PCR System (Roche, Basel, Switzerland) by adopting SYBR Green I Master Mix. The 20 μL reaction mixture comprised 3.0 μL cDNA, 1.0 μL (10 mM) of each primer, 10 μL Master Mix, and 5 μL PCR-grade water. Each reaction was performed in triplicate under the following conditions: 95 °C for 10 min, 40 cycles of 95 °C for 15 s, annealing at the specific temperature listed in Table S1 for 15 s, and 72 °C for 30 s. The relative expression level of each gene was calculated relative to the β-actin gene according to the 2^−ΔΔCt^ method.

### 2.6. Statistical Analysis

The biometric measurements and calculated data for two different culture modes and two sexes were separately compared, which divided the tested cases into four groups. Homoscedasticity and normality were tested for biometric measurements and calculated data, such as body weight, visceral coefficients, and biochemical parameters, which were determined by Levene’s test, and one-way analysis of variance (ANOVA) was conducted when *p* > 0.05. All data are presented as the means ± SEMs. All statistics on the transcriptome were performed on a bioinformatics analysis platform (http://www.omicshare.com/, (accessed on 15 March 2023)).

## 3. Results

### 3.1. Growth Performance

Male turtles grew faster than females in both pond-culture and co-culture modes, and the SGR was significantly higher in pond-cultured turtles than in co-cultured turtles (*p* < 0.05). There were no significant differences in the HSI of turtles of the same sex cultured in the two different modes (*p* > 0.05). The ISI was significantly lower for male pond-cultured turtles than for female pond-cultured turtles and co-cultured turtles (*p* < 0.05). There were no significant differences in the GSI of female turtles under different culturing modes (*p* > 0.05), while the GSI of male co-cultured turtles was lower than that of pond-cultured turtles (*p* < 0.05). LFI and MFI were higher in female turtles than in males, and they were both lower for co-cultured turtles than for pond-cultured turtles (*p* < 0.05) (Table 1). In addition, the difference in limb-fat color was visible; the limb fat of co-cultured turtles presented as bright yellow, while the limb fat of pond-cultured turtles presented as faint yellow or pale (Figure S1).

### 3.2. Physiological and Immune Indicators

**Figure 1:**
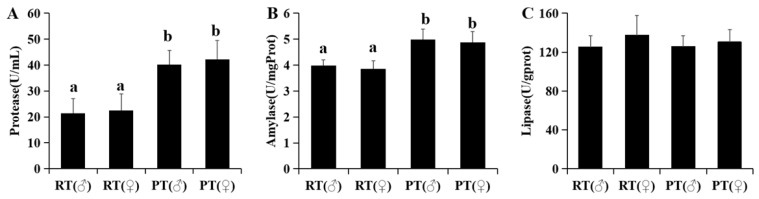
Activities of protease (**A**), amylase (**B**), and lipase (**C**) of pond-cultured turtles (PT) and co-cultured turtles (RT). Different letters on the bar indicate significant differences (*p* < 0.05).

The activities of protease and amylase in co-cultured turtles were significantly lower than those in pond-cultured turtles (*p* < 0.05), and there was no significant difference in lipase activity between the two different culturing modes (*p* > 0.05). There was no significant difference in digestive enzymes for turtles of different sexes in the same culturing mode (*p* > 0.05) (Figure 1).

The GLU of the co-cultured turtles was significantly lower than the GLU of the pond-cultured turtles (*p* < 0.05). There was no significant difference in the TG and TCHO of turtles under the two different culturing modes (*p* > 0.05), while there was a significant difference between male and female turtles (*p* < 0.05). The values of CRE, UA, and BUN were higher in co-cultured turtles than in pond-cultured turtles, especially in the male turtles (*p* < 0.05) (Table 2).

The AKP, ACP, and GOT were higher in co-cultured turtles of the same sex than in pond-cultured turtles, while the GPT was significantly higher in pond-cultured turtles (*p* < 0.05) (Table 3). There was no significant difference in the activities of SOD, GSH-PX, LZM, and C3 among turtles of the same sex cultured in ponds and paddy fields (*p* > 0.05), while CAT was higher in co-cultured turtles than in pond-cultured turtles of the same sex (*p* < 0.05) (Table 4).

### 3.3. RNA-Seq Data and Gene Expression Profiles

**Figure 2:**
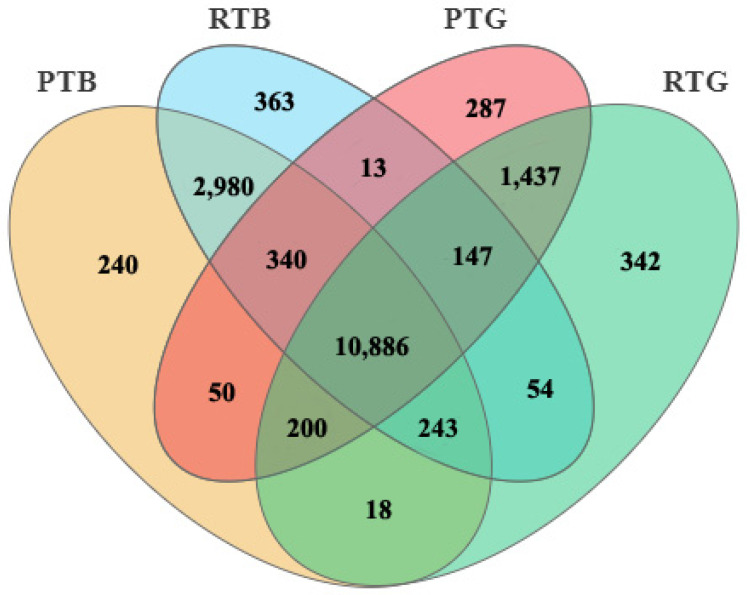
Common and unique transcribed genes in the brain (PTB and RTB) and gut (PTG and RTG) of pond-cultured turtles and co-cultured turtles.

The clean data numbered 56,045,690–76,173,154 for all samples, and the clean data rates were 99.19–99.44% (Table S2). In total, 25,222 genes were annotated in the samples, including 21,318 reference genes and 3904 novel genes in the present study (Table S3). For the brain, the number of common transcribed genes was 14,449, and the numbers of unique genes were 508 and 577 for pond-cultured turtles and co-cultured turtles, respectively. For the gut, the number of common transcribed genes was 12,470, and the numbers of unique genes were 690 and 657 for pond-cultured turtles and co-cultured turtles, respectively (Figure 2).

**Figure 3:**
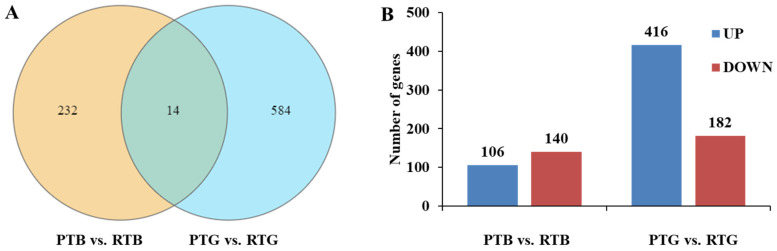
The number of DEGs (**A**) and upregulated and downregulated genes (**B**) in the brain and gut of pond-cultured turtles and co-cultured turtles.

The relative gene abundance in the brain and gut from turtles under different culturing modes was estimated based on fragments per kilobase of exon per million fragments mapped (FPKM). The FDR and log2-fold-change (fc) were used to screen for DEGs (FDR *<* 0.05 and |log2fc| > 1). There were 246 and 598 DEGs identified in the brain and gut from turtles cultured in the different modes, respectively (Figure 3A). The numbers of upregulated and downregulated genes in PTB vs. RTB were 106 and 140, respectively, while in PTG vs. RTG, the numbers of upregulated and downregulated genes were 416 and 182, respectively (Figure 3B). More details about the DEGs are listed in Supplementary Tables S4 and S5 for each group.

### 3.4. Functional Classification of DEGs

To investigate the biological importance of the DEGs, we performed a pathway enrichment analysis from the Kyoto Encyclopedia of Genes and Genomes (KEGG). The DEGs were mapped to six biological processes, including environmental information processing, cellular processes, genetic information processing, human diseases, metabolism, and organismal systems, in the KEGG_A_class. In PTB vs. RTB, 319 DEGs were identified in 160 pathways with a KEGG pathway annotation in the libraries, while in PTG vs. RTG, 1249 DEGs were identified in 260 pathways with a KEGG pathway annotation. In the PTB vs. RTB comparison group, most of the DEGs were mapped to signal transduction, signaling molecular and interaction, cancer:overview, and immune system at level 2. Metabolic pathways (ko01100, 14 genes), PI3K-Akt signaling pathway (ko04151, 9 genes), cytokine-cytokine receptor interaction (ko04060, 7 genes), and neuroactive ligand-receptor interaction (ko04080, 7 genes) were the pathways with the most genes.

**Figure 4:**
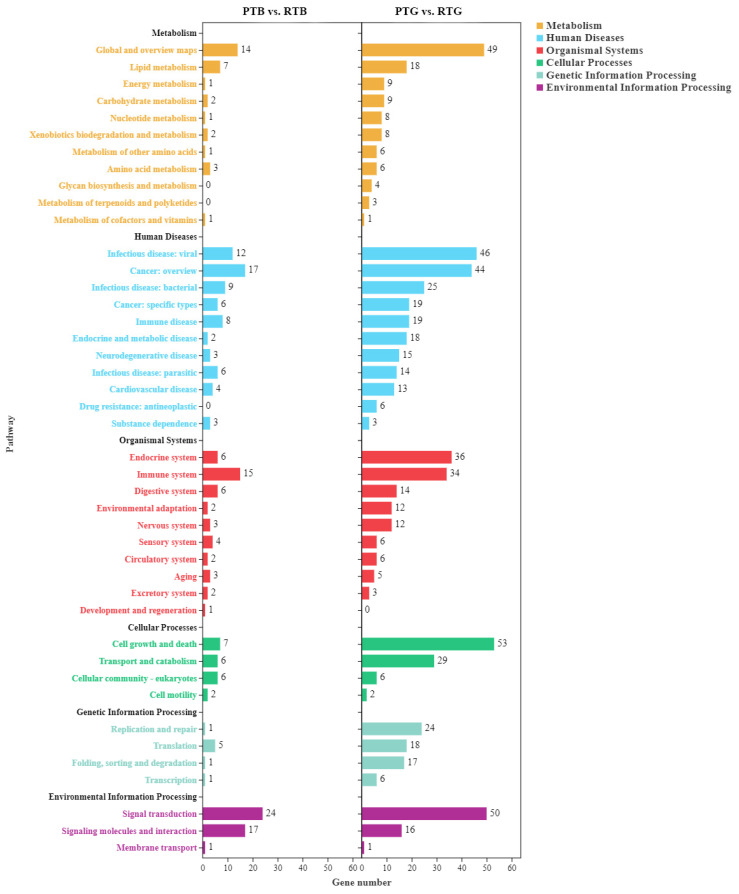
The enriched KEGG pathways of DEGs among Chinese soft-shelled turtles cultured in ponds and paddy fields.

**Figure 5:**
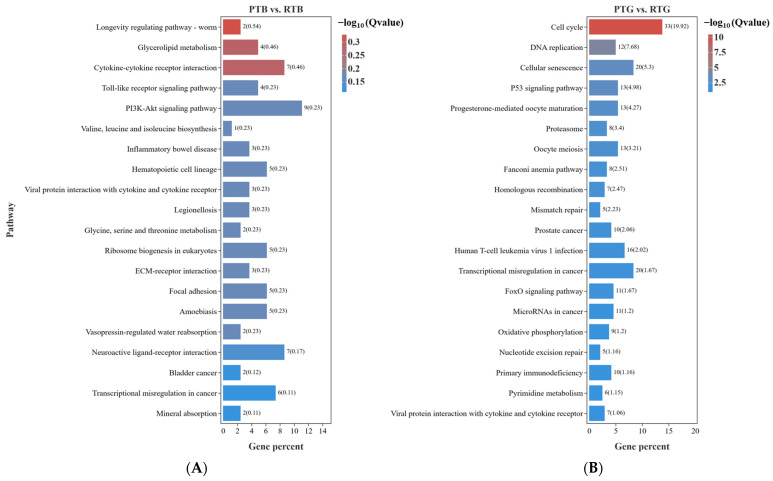
KEGG pathway enrichment analysis of differential genes in the two comparison groups. (**A**) The top 20 enriched pathways in the PTB vs. RTB comparison group. (**B**) The top 20 enriched pathways in the PTG vs. RTG comparison group.

In the PTG vs. RTG comparison group, most of the DEGs were mapped to cell growth and death, signal transduction, global and overview maps, infection disease, and cancer: overview at level 2 (Figure 4). Metabolic pathways (ko01100, 49 genes), cell cycle (ko04110, 33 genes), PI3K-Akt signaling pathway (ko04151, 21 genes), cellular senescence (ko04218, 20 genes), and transcriptional misregulation in cancer (ko05202, 20 genes) were the pathways with the most genes. We took the top 20 KEGG pathways for mapping according to the FDR and found differences between the two groups. In the PTB vs. RTB comparison, the top enriched pathways of the DEGs included the longevity regulating pathway, glycerolipid metabolism, cytokine-cytokine receptor interaction, Toll-like receptor signaling pathway, and PI3K-Akt signaling pathway. In PTG vs. RTG, the top enriched pathways of the DEGs included the cell cycle, DNA replication, cellular senescence, and the p53 signaling pathway (Figure 5). The significantly enriched KEGG pathways are listed in Tables S6 and S7 for PTB vs. RTB and PTG vs. RTG, respectively.

In PTB vs. RTB, more DEGs were related to environmental information processing and human diseases. Thirty-two DEGs were mapped to environmental information processing, including 10 upregulated genes such as DUSP4, CHRNA4, TNC, DGKH, TTI1, TRHR, and IGHV1-46 and 22 downregulated genes such as BDMD1, ITGA1, TNFSF15, EVC, ABCA9, P2RX5, EREG, CCL3, THBS1, and TSPO. Additionally, 35 DEGs were mapped to human diseases, including 28 downregulated genes such as SERPINB4, F2R, KLF2, MRC1, and TLR5 and 7 upregulated genes such as EGR1, H2B-VIII, FOS, and DGKH.

In PTG vs. RTG, 61 DEGs were mapped to environmental information processing, including 36 upregulated genes such as IGHVs, PLK1, IL22, STMN1, G4, IRF1, MMP3, and MTOR and 25 downregulated genes such as Hspa2, SGK1, dusp1-a, FOXO3, CCL3, HSP90AA1, ULK2, CXCL8, and PLA2G4F. Further, 92 DEGs were mapped to human diseases, including 29 downregulated genes such as ZNF620, NFKBIZ, Myo1e, ZNF251, and Mlxipl and 63 upregulated genes such as Kpna2, TOMM40, TOP2A, CCNB1, H2AFZ, CDK1, and TUBB4B. Moreover, 61 DEGs were mapped to metabolism, including 44 upregulated genes such as PPA1, DCTD, GGCT, ACOT12, and DCK, and 17 downregulated genes such as ASRGL1, ACOX2, HSD17B3, SAT2, GGT1, MOGAT2-b, and NUDT2.

### 3.5. Validation of DEGs by qPCR

**Figure 6:**
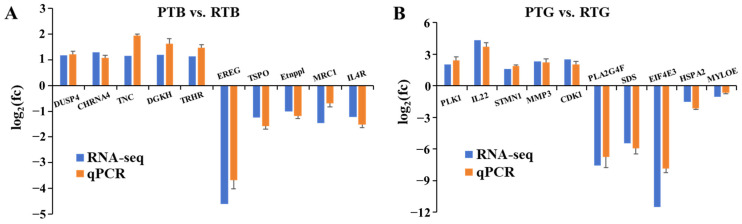
The DEGs expression ((**A**) brain; (**B**) gut) pattern obtained by using qPCR and RNA-seq.

PCR products were successfully amplified with all 20 specific primer pairs. For comparison with the results of the RNA-seq, the amplification efficiencies of the DEGs in the PTB vs. RTB groups and PTG vs. RTG groups were transformed by log2 (fc). The qPCR results were mainly consistent with the results of the RNA-seq, except for slight differences in expression levels (Figure 6), which confirmed the reliability of the RNA-seq data. Therefore, the growth- and immune-related genes annotated in this study could be useful references for future studies on the molecular mechanisms of Chinese soft-shelled turtles.

## 4. Discussion

Rapid growth is a highly desirable trait that enhances the profitability of food animal production and thus economic profit [28]. In the present study, the specific growth rate of pond-cultured turtles was significantly higher than that of co-cultured turtles without commercial feeds. The high growth rate of pond-cultured turtles was mainly attributed to the mass supply of commercial feed, and the lower growth rate of co-cultured turtles might mainly be due to scarce food sources, suggesting that paddy fields might not provide enough natural foods for the maximum growth of turtles and that they have the potential for rapid growth in paddy fields when more food is available. The slow growth of wild animals is mainly affected by an unstable natural food supply, poor living environment, energy constraints, competition, genetic factors, and self-protection mechanisms [29]. The co-cultured turtles were more similar to wild turtles in appearance and anatomy compared to intensively cultured turtles as well as in nutrition and flavor [3,26]. This reflects not only their physiological characteristics but also their ecological adaptation and evolutionary history [30,31].

Mesenteric and limb fat were important parts of weight gain for pond-cultured turtles. Excess mesenteric or visceral fat deposition is often accompanied by fatty liver and enteritis, which is the most common symptom in intensively farmed animals and has affected consumers’ desire to buy [32]. Limb fat is a type of adipose tissue that is stored in the limbs of some reptiles such as turtles [33]. It serves as an energy reserve that can be used during periods of food scarcity, hibernation, or reproduction; provides cushioning and lubrication for the joints; influences locomotion and performance; and plays a role in thermoregulation and water balance [34,35]. It is a dynamic tissue that can change in size and composition according to energy needs and metabolic status. Less fat deposition in co-cultured turtles might be an adaptive trait that evolved in response to the paddy field environment in this study. Low food intake and diversifying foods can prevent excessive fat accumulation in co-cultured turtles.

Aquatic animals are highly sensitive to the environment and have complex and sensitive responses against stress for self-protection and adaptation to different environments, including adjusting their physiological and biochemical characteristics [36]. Blood biochemical indices can better reflect the physiological metabolism of turtles [37]. Different habitats cause corresponding changes in biochemical indices, digestive and antioxidant enzymes, and immune factors for *P. sinensis*. Co-cultured turtles showed reduced GLU, while CRE, UA, and BUN were increased in the present study. The increased BUN, UA, and CRE levels in co-cultured turtles imply higher metabolic activities [38,39]. The increased BUN and UA values could cause an increase in osmolality, which may result in an increase in the prevention of water loss and adaptation to drought or hibernation [40]. This might help turtles adapt to paddy field environments with shallow water, especially during the drying stage. The higher BUN and UA levels of the co-cultured turtles indicate a shortage of nutrients and energy for the co-cultured turtles. The catabolism of protein and nitrogen compounds is strengthened and accompanied by an increase in UA formation when the energy balance is destroyed [41]. The stronger the catabolism, the more and the higher the UA formation, which can reduce the excessive oxidation and decomposition of protein in the process of vigorous exercise [42]. The protease and amylase levels were lower in co-cultured turtles than in pond-cultured turtles, which also indicates lower protein and carbohydrate intake for co-cultured turtles. The physiological response results also demonstrate that the turtles expended more energy on activities in the co-cultured mode with limited natural food, further leading to relatively lower weight gain but a healthier appearance.

A comprehensive analysis of growth performance, physiology, and transcriptomics can effectively evaluate the responses of *P. sinensis* to environmental factors [43,44,45]. Genes such as IGF1, IGF2, SLC27A2, GHRL, GHSR, MyoD1, WNT, and FGF have been demonstrated to be closely related to somatic growth [46,47,48,49]. The DEGs in the GH-IGF 1 axis genes and the Jak-STAT signaling pathway might play important roles in the growth differences of *P. sinensis* [50]. Meanwhile, signal transducers associated with the GH/GHR signaling pathway and IGF/IGFR signaling pathway were also found [43]. The upregulated TTI1 is important for mTOR stability and assembly of the mTOR complexes to maintain their activities [51,52]. Growth signaling pathways involving carbohydrate, protein, and lipid metabolism were found in the KEGG pathway analysis. In the present study, FGF, PLA, and ETS were significantly downregulated in the Ras signaling pathway of co-cultured turtles. MOGAT1 was downregulated, while DGKH was upregulated in glycerolipid metabolism, which could also be applied as a candidate biomarker to diagnose some diseases [53]. AMPK signaling inhibited gluconeogenesis and lipogenesis and promoted glycolysis to meet energy demand under high-ammonia conditions in *M. sinensis* [54]. Loannilli et al. (2020) found that FoxO1 balanced energy levels by increasing gluconeogenesis and decreasing glycolysis and lipogenesis [55]. In the present study, the AMPK-regulated transcription factors responsible for carbohydrate metabolism exhibited changes in the co-cultured groups: FoxO1 and CREB5 decreased, and the expression of PFKFB3 was subsequently downregulated. We also found that the level of GLU was reduced in the co-cultured turtle groups. These results indicate that GLU deficiency prompted the turtles to trigger glycolysis to fulfill the high energy requirement of the co-cultured turtles. All of these regulations are in accordance with the growth performance and metabolism of co-cultured turtles in terms of physiology.

Gene expression profiles also reveal differences in immunity in turtles cultured in different habitats [56,57]. PI3K/Akt pathways can be activated by stressors and regulate transcription, translation, proliferation, growth, and survival [58]. The genes EGF16, EREG, TNFSF15, IL4R, GHR, F2R, and ITGA in the PI3K/Akt pathways were significantly less expressed in co-cultured turtles. The cytokine-cytokine receptor interaction pathway is closely related to immunity, and it was demonstrated that cytokine-cytokine receptor interactions were activated in turtles after stress [59,60]. The downregulated number of cytokine and cytokine receptor genes were more intense in co-cultured turtles, and the significant downregulations included CCL4, CCL4L1, CCL4L2, CXCL8, IL4R, CSF3R, IL1R2, TNFSF15, and TNFSF10, which indicates a reduced immune response in co-cultured turtles. In the neuroactive ligand-receptor interaction pathway, the upregulated genes included GALR2A, TRHR, and CHRNA4, while the downregulated genes included F2R, P2RX5, and TSPO. TLR5, IL8, and CCL3 were downregulated in the Toll-like receptor signaling pathway. The higher expression of DUSP4 and TNC in co-cultured turtles indicated a lower risk of cancer [61,62]. All of these results indicate stable immunity and lower stress and disease risks in co-cultured turtles [63,64,65].

Adaptation can take a long time, while some behavioral and physiological acclimation may develop in the short term [66]. The hypothalamus plays a central role in the integrated regulation of energy homeostasis and body weight by activated neurons [67,68], while there were no significant differences in signaling molecules and receptors such as NPY, leptin, ghrelin, orexin, and melanocortin, which could modulate appetite or satiety in the present study [69]. The co-cultured turtles did not show a higher appetite than pond-cultured turtles, which indicates that the turtles in paddy fields had acclimated to the feeding habitat.

Rice paddies are consistent with the turtles’ wild habits, and co-cultured turtles present some natural behaviors similar to wild turtles, such as being more alert, aggressive, and ferocious [70]. Moreover, the co-culturing mode changed social environmental factors such as group size, composition, and hierarchy, which can affect the social behavior, communication, cooperation, competition, and aggression of both co-cultured turtles and pond-cultured turtles [71]. We determined the rice–turtle co-culture as a more ecological farm mode for *P. sinensis*. Enriching the environment for farmed turtles means providing them with more stimulation, diversity, and opportunities to express their natural behaviors, which improves their quality of life and reduces their suffering [72,73]. Meanwhile, co-cultured turtles in paddy fields have stable and predictable food sources due to artificial feeding, which can further meet their growth needs. However, turtles may prefer natural bait over compound feed or a switch between the two, depending on the availability and quality of the feeds [74]. Therefore, the feeding strategy should be further optimized based on nutrients demands, the abundance of natural food, feeding preference, feed efficiency, product quality, and environmental capacity.

## 5. Conclusions

*P. sinensis* in rice–turtle co-culturing with limited natural food tends to grow slowly. This study’s results in the physiological and biochemical indices such as the levels of GLU, CRE, UA, and BUN indicate low food intake and high metabolism in the co-cultured turtles. Most of the DEGs were mapped to environmental information processing, metabolism, and human diseases depending on transcriptome analysis for pond-cultured and co-cultured turtles. The comprehensive comparison of the growth and physiological and biochemical indices and the regulation of feeding and metabolism of *P. sinensis* demonstrated that rice–turtle co-culturing has positive effects on the aggression, physical health, antioxidant activity, and immunity of the turtles, while feeding strategies should be optimized to further enhance the economic and ecological benefits.

## Figures and Tables

**Table 1:** Growth performance and visceral coefficients of turtles subjected to different culturing modes.

Groups	Gender	FBW (g)	SGR (%/d)	HIS (%)	ISI (%)	GSI (%)	LFI (%)	MFI (%)
PT	♂	1298.7 ± 68.2 ^c^	0.60 ± 0.04 ^d^	3.48 ± 0.47 ^a^	1.38 ± 0.04 ^a^	1.65 ± 0.13 ^b^	6.59 ± 0.39 ^b^	1.27 ± 0.12 ^b^
	♀	1073.3 ± 46.2 ^b^	0.39 ± 0.03 ^c^	4.47 ± 0.32 ^b^	1.61 ± 0.11 ^b^	2.70 ± 0.30 ^c^	8.48 ± 0.51 ^d^	1.54 ± 0.10 ^c^
RT	♂	1029 ± 79.8 ^ab^	0.34 ± 0.01 ^b^	3.13 ± 0.25 ^a^	1.61 ± 0.15 ^b^	1.31 ± 0.14 ^a^	4.39 ± 0.33 ^a^	0.81 ± 0.07 ^a^
	♀	985.2 ± 54.1 ^a^	0.29 ± 0.01 ^a^	4.16 ± 0.25 ^b^	1.68 ± 0.09 ^b^	2.85 ± 0.32 ^c^	7.19 ± 0.68 ^c^	1.17 ± 0.11 ^b^

PT, pond-cultured turtles; RT, rice–turtle co-cultured turtles. data are presented as the means ± SEMs of three replicates. Different superscripts indicate significant differences (*p* < 0.05).

**Table 2:** Plasma biochemical parameters of co-cultured turtles and pond-cultured turtles.

Groups	GLU (mmol/L)	TG (mmol/L)	TCHO (mmol/L)	CRE (μmol/L)	UA (mg/L)	BUN (mmol/L)
RT (♂)	1.92 ± 0.62 ^a^	0.88 ± 0.11 ^a^	4.54 ± 0.18 ^a^	28.74 ± 3.33 ^b^	45.22 ± 3.55 ^c^	4.54 ± 0.23 ^c^
RT (♀)	5.73 ± 1.56 ^b^	5.6 ± 0.37 ^b^	8.53 ± 0.43 ^b^	39.04 ± 5.98 ^c^	26.92 ± 2.96 ^ab^	2.60 ± 0.14 ^a^
PT (♂)	9.93 ± 1.99 ^c^	0.85 ± 0.10 ^a^	4.66 ± 0.12 ^a^	20.60 ± 4.23 ^a^	28.75 ± 3.70 ^b^	2.91 ± 0.37 ^b^
PT (♀)	10.11 ± 2.84 ^c^	6.5 ± 0.52 ^b^	9.35 ± 0.56 ^b^	31.30 ± 5.50 ^b^	22.60 ± 3.54 ^a^	2.41 ± 0.16 ^a^

Different superscripts indicate significant differences (*p* < 0.05).

**Table 3:** Plasma metabolic enzymes of turtles cultured in ponds and paddy fields.

Groups	AKP (U/L)	ACP (U/L)	GOT (U/L)	GPT (U/L)
RT (♂)	1508.7 ± 52.9 ^d^	74.3 ± 5.3 ^b^	47.9 ± 5.5 ^b^	19.5 ± 3.0 ^a^
RT (♀)	1142.8 ± 60.5 ^b^	69.0 ± 6.0 ^b^	42.5 ± 3.9 ^b^	38.8 ± 4.4 ^d^
PT (♂)	1392.3 ± 59.8 ^c^	47.2 ± 5.9 ^a^	32.5 ± 4.2 ^a^	26.5 ± 3.5 ^b^
PT (♀)	1011.6 ± 61.4 ^a^	46.8 ± 4.6 ^a^	30.7 ± 5.1 ^a^	30.1 ± 3.9 ^c^

Different superscripts indicate significant differences (*p* < 0.05).

**Table 4:** Plasma antioxidant enzymes and immune indicators of turtles cultured in ponds and paddy fields.

Groups	CAT (U/mL)	GSH-PX (U/mL)	SOD (U/mL)	LZM (U/mL)	C3 (μg/mL)
RT (♂)	7.15 ± 1.03 ^b^	367.9 ± 10.2	100.5 ± 5.2	698.7 ± 55.8 ^ab^	5.21 ± 1.11
RT (♀)	6.64 ± 1.09 ^b^	361.8 ± 5.6	108.2 ± 9.9	637.5 ± 37.4 ^a^	4.96 ± 0.93
PT (♂)	4.43 ± 0.84 ^a^	370.0 ± 5.9	113.4 ± 11.3	728.9 ± 42.1 ^b^	5.86 ± 1.14
PT (♀)	4.09 ± 0.89 ^a^	357.3 ± 12.7	101.4 ± 8.6	700.3 ± 50.0 ^ab^	5.66 ± 1.26

Different superscripts indicate significant differences (*p* < 0.05).

## Data Availability

The data supporting the findings of this study are available within the article; there are no undisclosed data in this study.

## Supplementary Materials

The following supporting information can be downloaded at: https://www.mdpi.com/article/10.3390/ani14060962/s1, Table S1: The sequences of primers of qPCR for validation; Table S2: The RNA-seq data for all samples of brain and gut; Table S3: Gene number and comparison with reference genome; Table S4: The FPKM of DEGs of brain samples; Table S5: The FPKM of DEGs of gut samples; Table S6: KEGG pathway on DEGs of brain; Table S7: KEGG pathway on DEGs of gut; Figure S1: The anatomical figures of co-cultured turtles (A) and pond-cultured turtles (B).
