# Supplementary material for: Comparative Analysis of the Growth, Physiological Responses, and Gene Expression of Chinese Soft-Shelled Turtles Cultured in Different Modes
Source: Animals (Basel). 2024 Mar 20;14(6):962. doi: 10.3390/ani14060962 (PMC10967438; doi:10.3390/ani14060962)
Supplement: Supplementary file 1 [file animals-14-00962-s001.zip › Supplementary Table S1-S3.pdf]

Table S1 The sequences of primers of qPCR for validation

	Genes	Forward primer(5'-3')	Reverse primer(5'-3')	Annealing temperature
Brain	DUSP4	GGAGGATAACCACAAGGCT	TGTTTGACAAATTCAAAGG	51°C
	CHRNA4	GGAGAGTGGCGAATGGGTT	TTCCGTAATGAGCAGCAGG	58°C
	TNC	CATTCCTTCCACCACCGAT	GCCTGCCTTTCTCCACCAG	59°C
	DGKH	AAGGACCTGGGGATAACGA	CACCACACAAGAATGAAGA	52°C
	TRHR	GTGAAGATGGAGAACAGCA	AAGTGAAAGCCCAGACAAA	52°C
	EREG	ATCCTCTTTTTCCTCGTCG	TGTTTCTCTCCCCCTTCAC	54°C
	TSPO	CTGCTCCTTGCCGAACTCC	TGCCTCCCAAAAACCCTCC	61°C
	etnppl	ATTTCAACACGTTTGGAGG	TGTAGATGATATGCTGGGC	52°C
	MRC1	TATTTAGTCATCCGTTCCA	CAGGCTATTGTCTGCTTTG	50°C
	IL4R	GCCATTACTGATACTCCGA	AGTTGATTTTTTCCCCTTT	51°C
Gut	PLK1	GCCTCGGTTTTCCATTGCC	CTTCCTTTTCCTGGGTCCT	59°C
	IL22	GGGTTTTGGTGGTGGTTGT	CTCTTTTTGGAGGCATGGG	58°C
	STMN1	TGCCTACCTTCTTAGTCAT	CAGTTTAGCAGCCATTTGT	49°C
	MMP3	GGTGAAGGTATTGGTGGAG	CAGTTGGCAGATAGGTTGG	52°C
	Hspa2	CCTGGAAGGAGGGGGTAGC	CGAAGATGGTGTTGGTGGG	59°C
	Myo1e	ATGATTTCTGCTTATTTGT	AGTTTTCTTTGTGTTTTCT	44°C
	Kpna2	ATTTGTTGCCTTTTTGGGT	AATGGCGTCTATGGGTGGT	56°C
	CCNB1	GAGTTCAGGGCTTTGTGGT	GCAGTTTCGCTTCCTTTTT	55°C
	CDK1	GCACAGAGACTTAAAGCCT	AGTTGAGTAACGAGCGGAT	51°C
	PPA1	AGGTAATCAAAACCACTCA	GCATTTCAAACATCCATCC	50°C
	PLA2G4F	ACAGAACCCCTTACCCATC	CCAGTCCTGAACCAACCAT	54°C
	SDS	TTTTGGTATTGTTACTGCT	CTAATGTATGTCCTGATGG	45°C
	EIF4E3	GAGCAGAGAGATGGAGGAG	TGGGACAAAGACAGTGAGT	50°C
	β-actin	TGAGCTTCGTGTAGCACCTG	AGGATGGCATGGGGTAAAGC	58°C

Table S2 The RNA-Seq data for all samples of brain and gut

Samples	Raw Datas	Clean Data(%)	Adapter(%)	LowQuality(%)	polyA(%)	N(%)	Total_Mapped(%)
RTB-1	69035922	68597814(99.37%)	44128 (0.06%)	388750 (0.56%)	0 (0.00%)	5230 (0.01%)	59324601(86.98%)
RTB-2	69064872	68608364(99.34%)	39594 (0.06%)	411674 (0.60%)	0 (0.00%)	5240 (0.01%)	58957810(86.32%)
RTB-3	65401394	64955250(99.32%)	39880 (0.06%)	401224 (0.61%)	0 (0.00%)	5040 (0.01%)	56031997(86.58%)
RTG-1	58537250	58110414(99.27%)	36652 (0.06%)	385662 (0.66%)	0 (0.00%)	4522 (0.01%)	48992962(84.55%)
RTG-2	67690348	67212758(99.29%)	32326 (0.05%)	440588 (0.65%)	0 (0.00%)	4676 (0.01%)	55859949(83.38%)
RTG-3	56045690	55733704(99.44%)	31328 (0.06%)	276408 (0.49%)	0 (0.00%)	4250 (0.01%)	47246108(84.98%)
PTB-1	59091158	58728948(99.39%)	33896 (0.06%)	323766 (0.55%)	0 (0.00%)	4548 (0.01%)	50672127(86.76%)
PTB-2	63187932	62673172(99.19%)	34622 (0.05%)	477258 (0.76%)	0 (0.00%)	2880 (0.00%)	53966589(86.54%)
PTB-3	57070328	56660756(99.28%)	29012 (0.05%)	377356 (0.66%)	0 (0.00%)	3204 (0.01%)	49830753(88.56%)
PTG-1	65951916	65460004(99.25%)	34430 (0.05%)	452822 (0.69%)	0 (0.00%)	4660 (0.01%)	55517466(85.06%)
PTG-2	66024966	65582482(99.33%)	36664 (0.06%)	400732 (0.61%)	0 (0.00%)	5088 (0.01%)	54555586(83.44%)
PTG-3	76173154	75663626(99.33%)	41368 (0.05%)	462290 (0.61%)	0 (0.00%)	5870 (0.01%)	63511495(84.28%)

Table S3 Gene number and comparison with reference genome

Samples	Refer Genes	Sequenced Refer Genes(%)	Novel Genes	Sequenced Novel Genes(%)	Total Genes	Sequenced Total Genes(%)
All	24494	21318 (87.03%)	3904	3904 (100.00%)	28398	25222 (88.82%)
RTB-1	24494	18224 (74.40%)	3904	2999 (76.82%)	28398	21223 (74.73%)
RTB-2	24494	18173 (74.19%)	3904	2977 (76.26%)	28398	21150 (74.48%)
RTB-3	24494	18157 (74.13%)	3904	2953 (75.64%)	28398	21110 (74.34%)
RTG-1	24494	17089 (69.77%)	3904	2854 (73.10%)	28398	19943 (70.23%)
RTG-2	24494	17193 (70.19%)	3904	2866 (73.41%)	28398	20059 (70.64%)
RTG-3	24494	16960 (69.24%)	3904	2857 (73.18%)	28398	19817 (69.78%)
PTB-1	24494	18261 (74.55%)	3904	2935 (75.18%)	28398	21196 (74.64%)
PTB-2	24494	17955 (73.30%)	3904	2903 (74.36%)	28398	20858 (73.45%)
PTB-3	24494	17915 (73.14%)	3904	2810 (71.98%)	28398	20725 (72.98%)
PTG-1	24494	17392 (71.01%)	3904	2901 (74.31%)	28398	20293 (71.46%)
PTG-2	24494	16947 (69.19%)	3904	2761 (70.72%)	28398	19708 (69.40%)
PTG-3	24494	17385 (70.98%)	3904	2894 (74.13%)	28398	20279 (71.41%)
