# Supplementary figures and images for: Comparative Analysis of the Growth, Physiological Responses, and Gene Expression of Chinese Soft-Shelled Turtles Cultured in Different Modes
Source: Animals (Basel). 2024 Mar 20;14(6):962. doi: 10.3390/ani14060962 (PMC10967438; doi:10.3390/ani14060962)

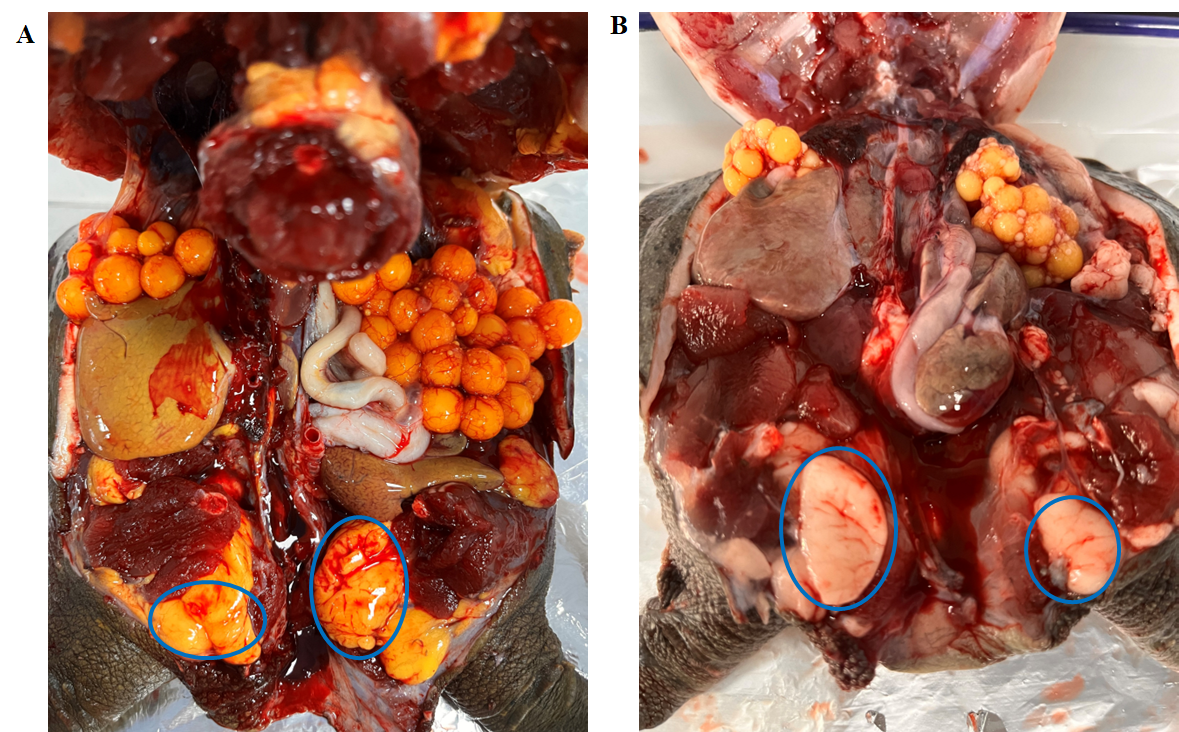

Supplement: Supplementary file 1 [file animals-14-00962-s001.zip › Figure S1 The anatomical figures of co-cultured turtle (A) and pond-cultured turtle(B), the circled is fat..tif]
